# Solid-Phase Optical Sensing Techniques for Sensitive Virus Detection

**DOI:** 10.3390/s23115018

**Published:** 2023-05-24

**Authors:** Elif Seymour, Fulya Ekiz Kanik, Sinem Diken Gür, Monireh Bakhshpour-Yucel, Ali Araz, Nese Lortlar Ünlü, M. Selim Ünlü

**Affiliations:** 1 Lunenfeld-Tanenbaum Research Institute, Mount Sinai Hospital, Toronto, ON M4P 1R2, Canada; elif.seymour@gmail.com; 2 Department of Biomedical Engineering, Boston University, Boston, MA 02215, USA; nese.lortlar@gmail.com; 3 Department of Electrical Engineering, Boston University, Boston, MA 02215, USA; fulyaekiz@gmail.com (F.E.K.); monir.b1985@gmail.com (M.B.-Y.); 4 Department of Biology, Hacettepe University, Ankara 06800, Türkiye; sinemdkn@hacettepe.edu.tr; 5 Department of Chemistry, Bursa Uludag University, Bursa 16059, Türkiye; 6 Department of Chemistry, Dokuz Eylül University, Izmir 35390, Türkiye; aliaraz272@gmail.com

**Keywords:** solid-phase optical biosensors, virus diagnostics, fluorescence-based sensors, surface plasmon resonance, optical resonators, interferometric biosensors, single-virus detection

## Abstract

Viral infections can pose a major threat to public health by causing serious illness, leading to pandemics, and burdening healthcare systems. The global spread of such infections causes disruptions to every aspect of life including business, education, and social life. Fast and accurate diagnosis of viral infections has significant implications for saving lives, preventing the spread of the diseases, and minimizing social and economic damages. Polymerase chain reaction (PCR)-based techniques are commonly used to detect viruses in the clinic. However, PCR has several drawbacks, as highlighted during the recent COVID-19 pandemic, such as long processing times and the requirement for sophisticated laboratory instruments. Therefore, there is an urgent need for fast and accurate techniques for virus detection. For this purpose, a variety of biosensor systems are being developed to provide rapid, sensitive, and high-throughput viral diagnostic platforms, enabling quick diagnosis and efficient control of the virus’s spread. Optical devices, in particular, are of great interest due to their advantages such as high sensitivity and direct readout. The current review discusses solid-phase optical sensing techniques for virus detection, including fluorescence-based sensors, surface plasmon resonance (SPR), surface-enhanced Raman scattering (SERS), optical resonators, and interferometry-based platforms. Then, we focus on an interferometric biosensor developed by our group, the single-particle interferometric reflectance imaging sensor (SP-IRIS), which has the capability to visualize single nanoparticles, to demonstrate its application for digital virus detection.

## 1. Introduction

Viral infections can pose a serious threat to public health, as demonstrated by the recent COVID-19 pandemic, which was caused by a novel coronavirus, SARS-CoV-2, infecting 676 million people and causing 6.8 million deaths worldwide as of March 2023 [1]. Human history has witnessed several outbreaks caused by viruses such as the plague [2], cholera [3], flu [4], and HIV [5]. The 1918 Spanish flu pandemic that was caused by the H1N1 virus was the deadliest flu pandemic in recorded human history, with an estimated 50 million deaths worldwide [6]. The recent COVID-19 pandemic and the constant emergence of new outbreaks such as Ebola, Zika, RSV, and monkeypox highlighted the urgent need for sensitive and high-throughput viral diagnostic techniques. It is crucial to detect viral infections in a fast and sensitive fashion to enable effective infection control and improve health outcomes [7].

Over the years, several approaches have been used for clinical virus diagnostics including virus isolation in culture, enzyme-linked immunosorbent assay (ELISA), and polymerase chain reaction (PCR) [8]. Virus culture, referred to as the ‘old gold standard’, is performed by growing viruses in cell culture and takes 2–12 days [9]. In addition to being time-consuming, virus isolation in culture can be difficult and expensive [10]. Serological diagnostics using ELISA is based on the detection of antibodies produced by the immune system as a result of the viral infection. This indirect detection approach has its limitations. The antibody response may not be strong enough to allow detection in the early stages of the infection, or a weak serological response in certain patients can lead to false negative results [11]. Quantitative or real-time PCR (RT-PCR) is the current gold standard for molecular diagnostics of viruses in clinical settings [12]. This technique involves the amplification of certain regions in the viral genome using specific primers and other reagents such as enzymes and nucleotides. Although RT-PCR is an extremely sensitive detection technique, it requires highly automated equipment for nucleic acid extraction, expensive thermocyclers for the amplification reaction, and skilled users to operate these instruments. Moreover, as revealed during the peak times of the COVID-19 pandemic, reagent and trained personnel shortages and insufficient equipment and infrastructure led to long turnover times, overwhelming the laboratories and delaying the results [13]. In an effort to overcome these limitations, alternative virus-detection methods, either nucleic-acid- or antigen-based, were developed for rapid point-of-care (POC) diagnostics and received emergency use authorization (EUA) from the U.S. Food and Drug Administration (FDA) during the COVID-19 pandemic [14]. These include isothermal amplification-based and CRISPR (Clustered Regularly Interspaced Short Palindromic Repeats)-based nucleic-acid-detection platforms as well as antigen tests employing immunofluorescence or immunochromatographic lateral-flow assays [15,16,17,18]. However, despite providing quick results at the POC, these tests have a limited throughput and variable sensitivities [19]. Due to the continuing need for rapid, sensitive, and high-throughput virus detection platforms, there has been considerable effort toward developing biosensors for viral diagnostics applications [20].

An ideal viral diagnostic platform should be highly sensitive, fast, high-throughput, and easy to use and require minimal sample processing and temperature-sensitive reagents. Since the first biosensor was developed in 1956 by Leland C. Clark for detecting oxygen, there have been tremendous advancements in the biosensor field [21]. Biosensors consist of a biological sensing element for specific analyte binding and a transducer system to convert the binding events to a measurable signal. Biosensors can use electrical, optical, or mechanical transduction mechanisms to convert the changes induced by the biological interactions on the sensor to an observable output, which then can be correlated with the biological binding interactions. Biosensors hold great potential for being used as viral diagnostic tools at the POC owing to the advantages they offer such as simple workflows, cost-effectiveness, portability, and rapid answers. Moreover, the integration of microarray and microfluidics technologies into biosensors enabled multiplexed detection and the use of smaller sample and reagent volumes compared to the laboratory techniques such as ELISA.

In this review, we focus on solid-phase optical sensors for viral diagnostics applications. Optical sensors have advantages compared to other transduction mechanisms due to their direct detection capability and minimal dependence on environmental conditions [22]. For viral diagnostic applications, an optical biosensor can be used to determine the presence of an infection by detecting viral antigens, whole viruses, viral nucleic acids, or an individual’s antibody response in biological samples. In solid-phase biosensors, capture agents against one of these analytes are immobilized on the sensor surface. The solid-phase optical biosensors that are covered in this review include fluorescence-based optical sensors, colorimetric biosensors, surface plasmon resonance (SPR), surface-enhanced Raman scattering (SERS), optical resonators, and interferometry-based platforms including single-particle interferometric reflectance imaging sensor (SP-IRIS), a label-free biosensor developed by our group.

## 2. Solid-Phase Optical Sensors and Their Applications in Virus Detection

**Figure 1:**
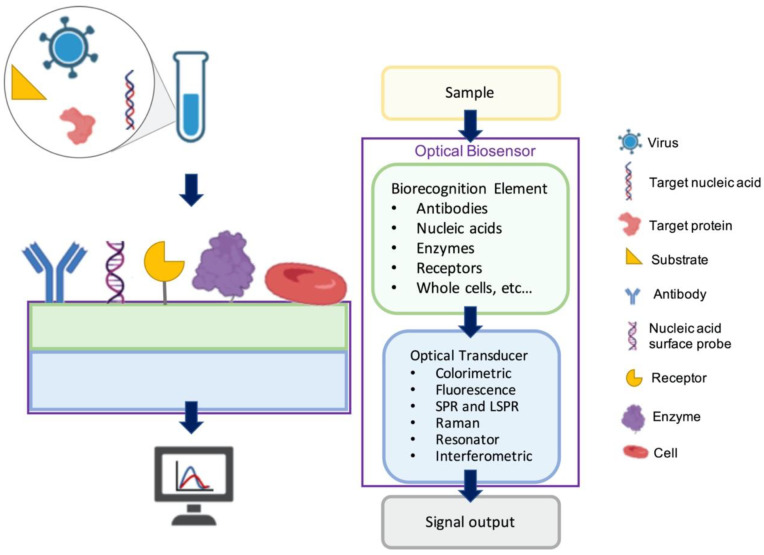
A general illustration of optical biosensors.

Optical biosensors measure the optical signals as the changes in the optical properties and characteristics on the transducer surface in the case of an interaction of the immobilized biorecognition element with the measured substance [23,24,25]. Figure 1 shows a general illustration of an optical biosensor. The optical biosensor consists of an optical source, a transduction platform and an optical detector. Optical biosensors can use different types of biorecognition elements such as antibodies, aptamers, peptides, nucleic acids, peptide nucleic acids, proteins, enzymes, or whole cells on the transducer surface, which is designed to bind with the target substance specifically [26,27,28,29,30]. The optical transducer is integrated closely with the biosensing element. The optical biosensors are classified based on their transducers and can also be classified based on their dependence on a label for signal generation as label-based [31,32] and label-free [33,34,35,36,37]. In label-based biosensors, the detected signal originates from the label, such as a fluorophore or chromophore, conjugated to the detection molecule, which binds to the captured target. In contrast, in label-free biosensors, the signal produced by the interaction between the target and the biorecognition element is measured directly. In this section, we discuss a selection of surface-based optical biosensor technologies for the detection of viruses with an emphasis on their principles of detection and their applications in sensitive viral diagnostics.

### 2.1. Fluorescence-Based Optical Sensors for Virus Detection

Fluorescence-based optical biosensors employ fluorescent labels to produce the optical signal, which results from the binding of the labeled detection molecule to the capture probe and analyte complex on the transducer [23,38,39]. They are widely used in assay development owing to numerous commercially available fluorescent labels, uncomplicated labeling methods, multi-color fluorophores for multiplexed assays, fast response times with localized fluorescence signal, high temporal resolution, and sufficient detection sensitivity [40]. These advantages of fluorescence optical biosensors are desirable for the detection of viruses and biological molecules [41,42,43,44]. However, certain limitations such as fluorophore blinking, photobleaching, and insufficient detection limits for some target molecules make fluorescence-based optical biosensors less applicable in certain applications, for instance, in the detection of low-abundance nucleic acids [45]. Moreover, nonspecific binding of the fluorescent labels to other components in the sample media remains an issue in fluorescence detection systems [46]. Additionally, irreversible photobleaching of the fluorescent label restrains the observation time, hence affecting the reliability of the test [47].

Fluorescence-based optical biosensor technologies are commonly compared with ELISA due to the similarities in the detection method, application, the use of common fluorescence labels, and ELISA’s widespread use and reliability. ELISA requires a laboratory environment, an intricate process, sophisticated equipment, and trained personnel, making it a less-ideal choice for low-resource settings [48,49]. On the other hand, fluorescence-based optical sensors stand out by offering point-of-care (POC) testing and cost-effectiveness. With the advances in technology, smartphones have become more integrated into the recent designs of fluorescence-based POC optical biosensor platforms. Smartphones can be used to visualize labeled viruses and fluorescent nanoparticles by incorporating them into POC devices. Biosensors using smartphones for monitoring either take advantage of the phone’s built-in sensors, such as the camera, magnetic sensor, and ambient-light sensor, or use external sensor modules connected via wired or wireless connections to the integrated diagnostic system. Smartphone-based virus-detection systems are not as sensitive as gold-standard diagnostic methods. However, these systems are portable and scalable, and therefore, they have good prospects for the development of an accessible POC for viral disease surveillance and management [50].

Here, we present a number of fluorescence-based biosensor applications for virus detection.

**Figure 2:**
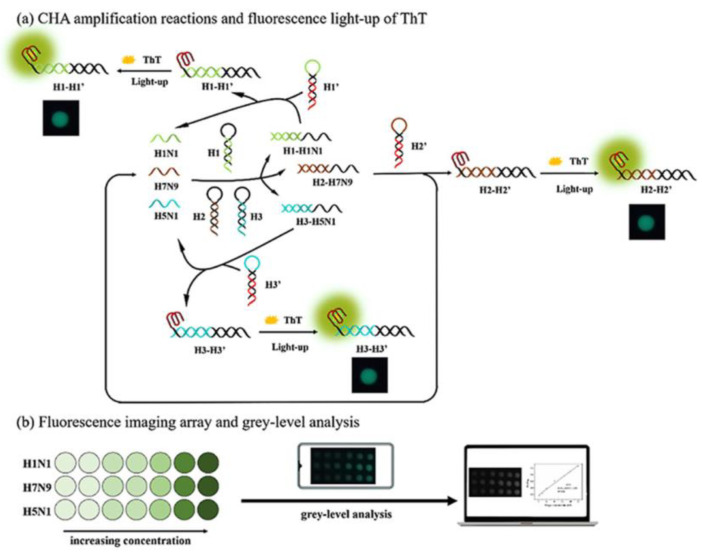
Multiplex imaging array design for rapid diagnosis of trace avian influenza virus (AIV) using DNA biomarkers based on (**a**) CHA amplification reactions and (**b**) fluorescence imaging array and gray-level analysis. Reproduced with permission from [51]. Copyright © 2021 Elsevier.

A multiplex imaging array was developed for the rapid and low-cost diagnosis of trace avian influenza virus (AIV) using DNA biomarkers by Jiang et al. (Figure 2) [51]. They detected three subtypes of AIV DNA biomarkers (H1N1, H7N9, and H5N1) simultaneously using fluorescence imaging and gray-level analysis. They utilized a smartphone for imaging the output and completed detection in 20 min. They employed catalytic hairpin assembly (CHA) amplification reactions and utilized thioflavin T, a specific G-quadruplex fluorescence probe for labeling. CHA is a non-enzymatic amplification technique that is commonly used for the detection of various nucleic acids with high sensitivity. In CHA, two complementary nucleic acid hairpins are used, which undergo a cascade of very quick hybridization events and, at the end, the final double helices are produced. These helices can generate fluorescence signal in a short time. In the study, the authors reached detection limits of 136 pM, 141 pM, and 129 pM for H1N1, H7N9, and H5N1, respectively. Moreover, the array sensors exhibited excellent anti-interference among the different subtypes and good mismatch discrimination in real samples. Such a system can easily be applied for the early detection of disease diagnostics in low-resource settings.

**Figure 3:**
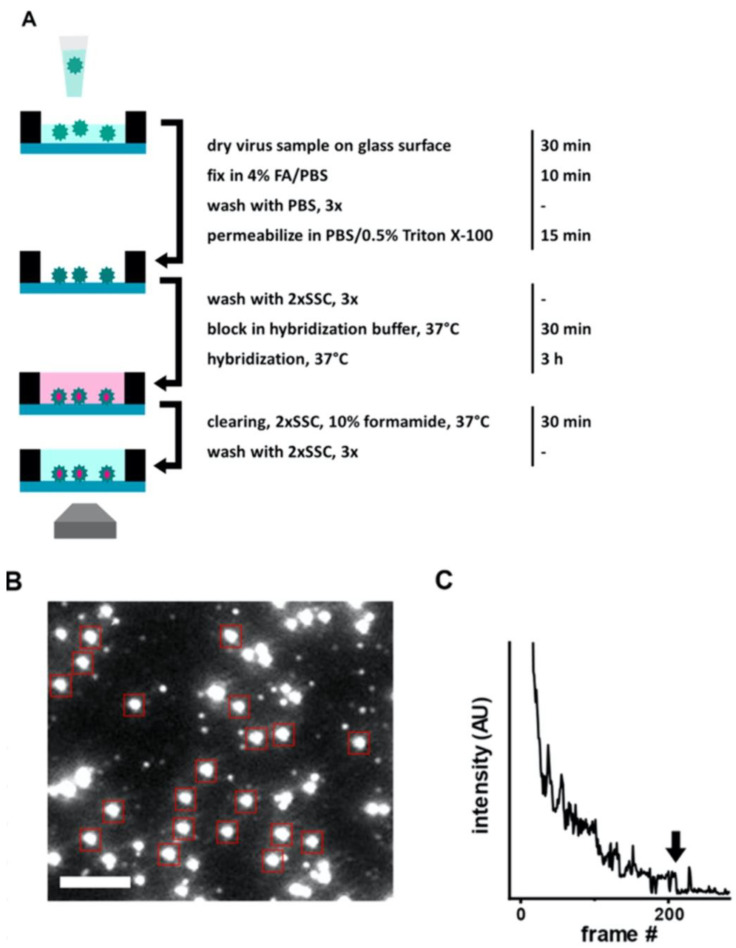
(**A**) Illustration of FISH-based virus detection. (**B**) Image of an array after staining with 48 fluorescent hybridization probes for influenza A/WSN/33 virus particles (10^6^ PFU/mL). Red boxes point diffraction-limited, isolated particles which were analyzed with stepwise photobleaching. Scale bar 3 μm. (**C**) Representative photobleaching curve for obtaining the average number of probes bound to a virus particle in the FISH assay. Arrow shows the final bleaching step used for determining the average intensity of a single probe on a particle. Reproduced with permission from [52]. Copyright © 2021 Springer Nature.

Another rapid viral detection and identification study was performed by Hepp et al. for detecting influenza virus, avian infectious bronchitis virus, and SARS-CoV-2 specifically and quantitatively in approximately 20 min (Figure 3) [52]. They used a fluorescence *in situ* hybridization (FISH) protocol, specifically a rapid viral FISH protocol (rvFISH), where they used fluorescence microscopy to spatially detect and quantify DNA and RNA inside fixed cells and tissues using complementary and fluorescently labeled oligos. They used a single-molecule TIRF microscope to image immobilized and stained virus particles, where they count the bound FISH probes by stepwise photobleaching (Figure 3C). They obtained Figure 3C by averaging the decrease in fluorescence intensity of the last bleaching step over several molecules, then obtained the average fluorescence intensity related to the single fluorophore of each FISH probe on the virus particle. They were able to detect influenza particles and infectious bronchitis virus (IBV), an avian coronavirus, down to a concentration of 10^5^ PFU/ mL and 10^2^ PFU/ mL, respectively, in a 20 min assay.

**Figure 4:**
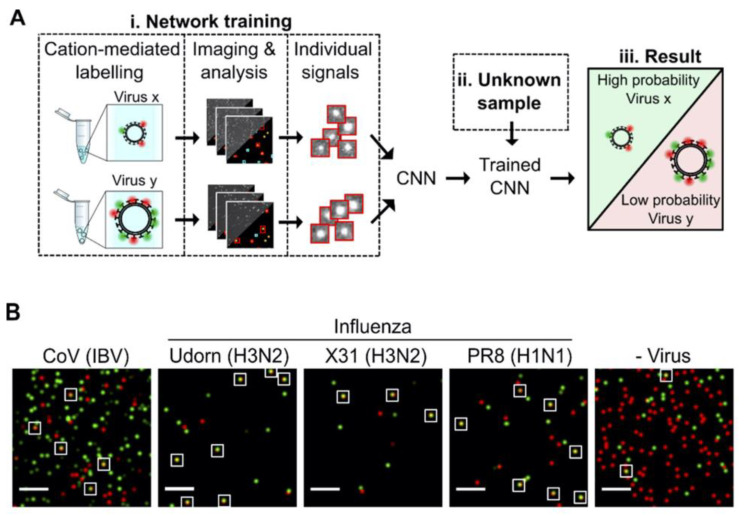
(**A**) Illustration of how the viruses are labeled and imaged to identify and classify different viruses using trained convolutional neural network (CNN). (**B**) Sample field of views for CoV (IBV), two strains of H3N2 influenza (A/Udorn/72 (Udorn), and A/Aichi/68 (X31)), an H1N1 influenza strain (A/PR8/8/34 (PR8)) detection, and a negative control. White boxes were used to point out merged red and green localizations as an example of colocalization. Scale bar, 10 μm. Reproduced with permission from [53]. Copyright © 2022 American Chemical Society.

Shiaelis et al. present a methodology for virus detection and identification that uses a convolutional neural network (CNN) to distinguish between microscopy images of fluorescently labeled intact different viral particles (Figure 4) [53]. They utilized single-particle fluorescence microscopy and deep learning. The assay successfully performed labeling, imaging, and virus identification in less than 5 min and did not require any lysis, purification, or amplification steps. They carried out two clinical tests using 155 patient samples in total, which provided high overall sample accuracies of 98.0% and 97.1%.

**Figure 5:**
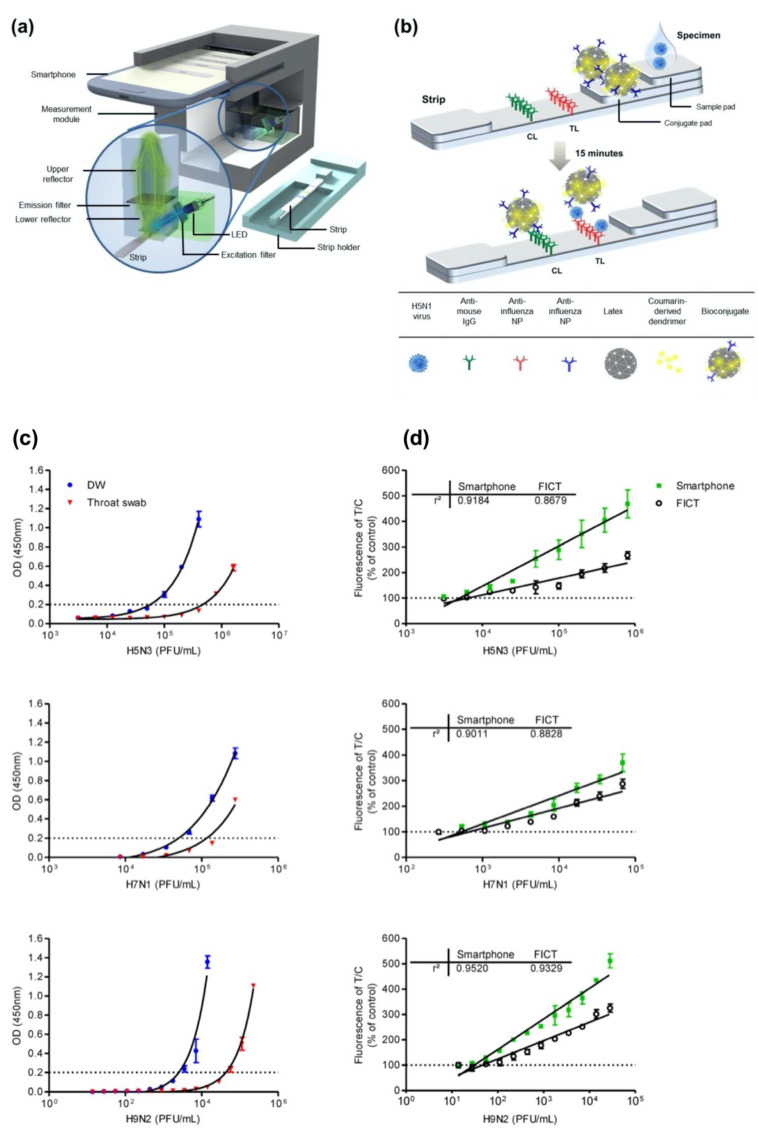
(**a**) Illustration of the fluorescence-detector design using a smartphone and fluorescent lateral-flow strip. (**b**) Illustration of the fluorescence lateral strip for influenza A and the components for lateral-flow strip design. A nitrocellulose membrane as the base was decorated with anti-influenza A nucleocapsid (NP) antibody on the test line (TL) and anti-mouse IgG on the control line (CL). Bioconjugate-AI virus complex is captured by the anti-influenza NP on the TL. On the other hand, the unreacted bioconjugates are captured by the anti-mouse IgG on the CL. The fluorescence intensity is measured using the smartphone-integrated diagnostic device. (**c**) ELISA quantification results and (**d**) table-top fluorescent immunoassay strip reader (FICT) and the smartphone-based detector quantification results of H5N3, H7N1, and H9N2 viruses containing samples in distilled water (DW) or a normal throat swab. The normalized fluorescent values of TL/CL for each negative control were shown with horizontal dotted lines. Each data point represents mean ± SD (*n* = 3). Reproduced with permission from [54]. Copyright © 2016 Ivyspring International Publisher.

Moreover, Yeo et al. have demonstrated a field-level fluorescent lateral flow immunoassay that was combined with a smartphone-based fluorescent diagnostic device with an efficient reflective light-collection module for the detection of avian influenza (AI) (H5N1, H5N3, H7N1, and H9N2) (Figure 5a,b) [54]. The fluorescence light efficiency was improved with the use of latex beads along with the coumarin-derived dendrimer-based fluorescent as anti-influenza nanoparticles. The lowest detectable virus titers were found as 6.25 × 10^3^ PFU/mL for H5N3, 5.34 × 10^2^ PFU/mL for H7N1, and 5.23 × 10^1^ PFU/mL for H9N2 in throat-swab samples. The diagnostic test system was also compared with sandwich ELISA in the quantification of all the virus samples both in distilled water (DW) and normal throat swabs. The results showed that the proposed smartphone device had an improved diagnostic performance compared to ELISA and the table-top version. In addition, clinical validation of a smartphone-based diagnostic device with H5N1-infected patient samples was completed within 15 min with a sensitivity of 96.55% (28/29) [95% confidence interval (CI): 82.24 to 99.91] and a specificity of 98.55% (68/69) (95% CI: 92.19 to 99.96) (*p* < 0.0001).

### 2.2. Colorimetric Biosensors for Virus Detection

Colorimetry-based biosensors allow visual detection via a change in color detected with the naked eye or simple, low-cost, and portable optical detectors. These features make them proper candidates to fabricate POC devices that can be used for rapid and cost-effective virus detection [55]. They employ a simple platform with a quick response and fair sensitivity and selectivity [56]. In colorimetry-based solid-phase biosensors, on the sensor surface, which is usually a simple test strip, when the sample solution is introduced, a ligand–target complex is formed on the solid support. This complex results in a shift in color that can be easily observed for quantitative measurements. With the recent advancements in nanotechnology, the sensitivity of colorimetric detection systems has been improved by using various functional nanomaterials such as metal and metal oxide NPs, quantum dots, graphene, and derivatives [57]. In the NP-based approach, colloidal NPs that change color during aggregation or dispersion are conjugated with the biosensing element. Plasmonic-based colorimetric biosensors benefit from the localized surface plasmon resonance (LSPR) extinction coefficient in the visible range of noble metal NPs such as gold NPs (AuNPs). Binding events between the analyte and the AuNP-conjugated bioreceptor cause visible color change to show the presence of the virus [58]. NP-based colorimetric sensors can be used in a wide range of virus sensing applications. Khoris and coworkers designed an immunoassay-based sensing technique that detected hepatitis E virus (HEV) in real-time using Ag-decorated AuNPs. Anti-HEV IgG antibodies were conjugated to AuNPs and *in situ* silver deposition was achieved on the surface of antibody–AuNP conjugates as a signal-amplification strategy. The virus particles were entrapped by the utilized nanocomposites whereas 3,3′,5,5′-tetramethylbenzidine (TMB) and H_2_O_2_ were added to decompose back the Ag shell to Ag^+^. After the addition of TMB-H_2_O_2_, based on the obvious color change, the concentration of HEV was quantified and real-time monitoring of HEV in a real sample was realized [59].

**Figure 6:**
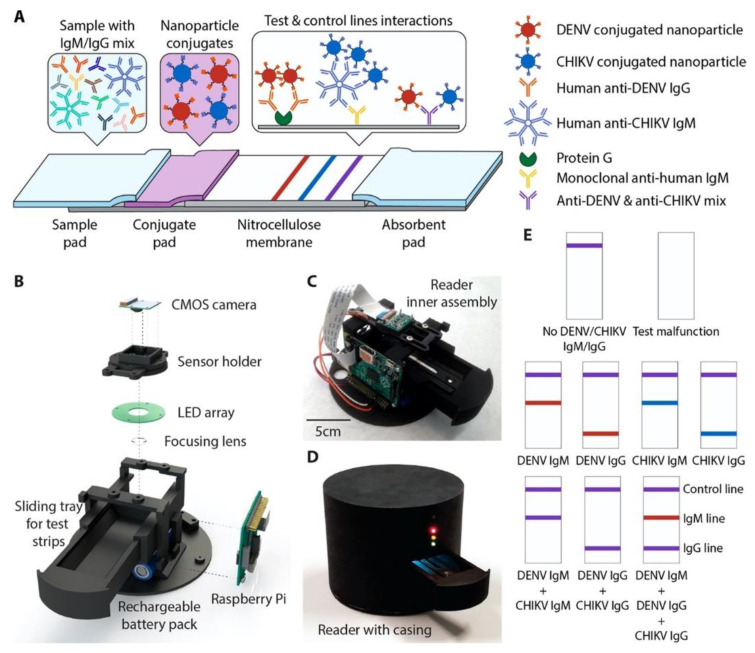
(**A**) A 4-plex colorimetric lateral-flow test strip and an optical readout device. Red and blue nanoparticle conjugates in the conjugate pad bind to DENV and CHIKV antibodies in the sample, respectively. Nanoparticle-IgG complexes are captured by the first test line (red), and nanoparticle-IgM complexes are captured by the second test line (blue), causing a color change. When both DENV and CHIKV antibodies of the same isotype are present, a mixture of red and blue (purple) appears at the test line. Unbound nanoparticle conjugates are captured at the control line (purple). (**B**) Parts of the optical reader. (**C**) Inner view of the optical reader. (**D**) Fully assembled version of optical reader with lightproof casing. (**E**) Examples showing the color development on the test strip for various cases. Reproduced with permission from [61]. Copyright © 2019 American Chemical Society.

Paper-based lateral flow immunoassays (LFIAs) as POC devices are widely used for early disease diagnostics. Despite their widespread use, they are often limited due to insufficient sensitivity for the required sample sizes and short time frames of testing. Loynachan et al. designed a highly sensitive, serum-stable, paper-based, and nanoparticle catalyst-labeled LFIA for the detection of a viral capsid protein, p24, one of the earliest and most conserved biomarkers of HIV. They used porous platinum core-shell nanocatalysts (PtNCs), and then explored the application of antibody-functionalized PtNCs with high-affinity and -specificity modified nanobodies toward p24. They established the key larger-nanoparticle-size regimes needed for efficient amplification and performance in LFIA [60]. In another study, Wang et al. designed a rapid diagnostic platform integrated with a low-cost reader and a multicolor four-plex immunoassay to detect and distinguish between dengue virus (DENV) and chikungunya virus (CHIKV) IgM/IgG [61]. The developed platform employs a unique color-mixing encoding and quantitative readout strategy while using an optical reader designed to minimize the variation in color detection. The assay uses red- and blue-colored 400 nm latex nanoparticles conjugated to DENV (red) and CHIKV (blue) envelope proteins. When DENV and/or CHIKV IgM/IgG antibodies are present in the sample, they bind to the corresponding NPs, which are then captured on the appropriate line of the test strip (Figure 6). This platform provides a consistent multiplexed detection of dengue and chikungunya IgM/IgG antibodies in human clinical samples within 30 min. The multiplex assay requires low sample volumes and has the ability to test four samples simultaneously, which makes the rapid diagnostic platform a great candidate to be used in resource-limited settings.

### 2.3. Virus Detection with Surface Plasmon Resonance and Localized Surface Plasmon Resonance

Surface plasmon resonance (SPR) is defined as an electromagnetic (EM) phenomenon depending on the collective resonant oscillations of free electrons and incoming protons passing through a metal-dielectric interface. The working principles of SPR-based optical sensors depend on the detection of changes in the refractive index that arise on the dielectric surface near the metal layer [62,63]. The properties of this metal layer strongly influence the SPR response that is generated according to the refractive index change. The metals that have conduction-band electrons, such as gold, silver, aluminum, and copper, show the ability to resonate at an appropriate wavelength with the incident light. Gold is the most preferred metal film due to its chemical stability and sensitivity for sensing applications [64]. A typical SPR-based sensor consists of three main components: (i) the immobilized recognition element, (ii) the prism of light, and (iii) the analyte [65]. The recognition molecule is immobilized onto the gold surface of the sensor chip. After surface functionalization, a sample solution containing the analyte is passed across the chip surface. The incident light passing through the prism excites the electrons of the metal film to form a surface plasmon. As the incident light reaches the medium at various angles, the photons are absorbed by the plasmon wave at a specific angle, the critical angle, which is affected by the refractive index of the medium. When an analyte binds to the immobilized recognition element, due to the mass accumulation on the immobilized layer, the refractive index of the medium near the chip surface changes, which shifts the critical angle for the immobilized molecule [66,67,68]. Thus, any physical change that causes a refractive index change can be monitored without labeling in real time. SPR-based sensing techniques offer good potential for rapid and POC detection of viruses due to their sensitive and label-free detection mechanisms. Antibodies against viral antigens are used as bio-receptors to capture viral proteins and intact viruses on the sensor chip surface. Additionally, artificial recognition sites obtained using molecular imprinting or laboratory-made capture molecules, such as DNA and RNA aptamers, are used to capture several viruses [69]. SPR sensing technology, which is highly accurate in detecting biomolecular interactions, also offers various advantages such as label-free monitoring, rapid and sensitive detection, and the ability to miniaturize for on-site monitoring [70]. However, to succeed in the early diagnosis of viruses using the SPR method, further enhancements in the selectivity and sensitivity are still required [69]. Here, different SPR-based techniques that can detect viruses are discussed.

**Figure 7:**
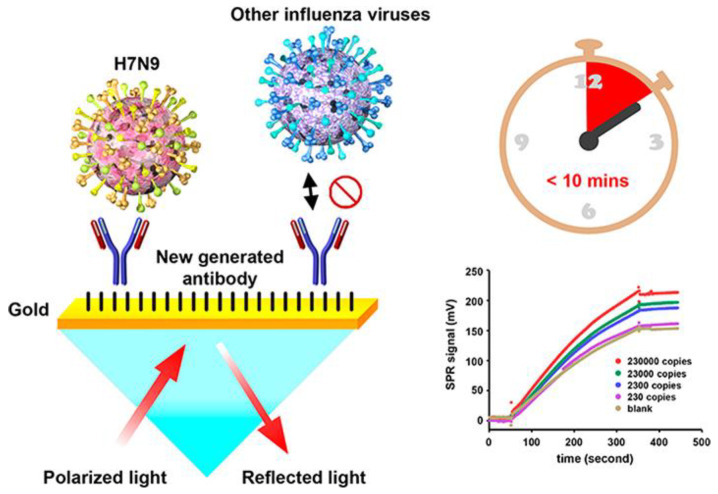
The schematic immobilization of antibody H7-mAb to the reaction spot of the SPR chip and detection of the viruses using the IM-SPR sensor. The bottom graph shows the SPR signal for different concentrations of the H7N9 virus in PBS, spanning a range between 2.3 × 10^2^ and 2.3 × 10^5^ copies/mL. Reproduced with permission from [71]. Copyright © 2018 American Chemical Society.

Chang et al. developed an intensity-modulated surface plasmon resonance (IM-SPR) biosensor integrated with a newly generated monoclonal antibody that enables the rapid and sensitive detection of a new strain of avian influenza A H7N9 virus that emerged in China in 2013. [71]. The novel antibody displays high specificity for the H7N9 virus compared with other human influenza viruses. They experimentally reported a detection limit of 144 copies/mL using the proposed approach for the H7N9 virus, which is a 20-fold increase in sensitivity compared with homemade target-captured ELISA. They reported a less than 10 min assay time. 10 μg/mL of the capture antibody H7-mAb was covalently immobilized to the reaction spot of the SPR chip through mixed self-assembled monolayers of 11-mercaptoundecanoic acid and 6-Mercapto-1-hexanol (1:9) via the amine coupling protocol (Figure 7). The results demonstrated that the simple SPR-based technology was successfully used in sensitive H7N9 virus detection. They reported that the proposed SPR system can be used in the implementation of other emerging virus-detection platforms.

**Figure 8:**
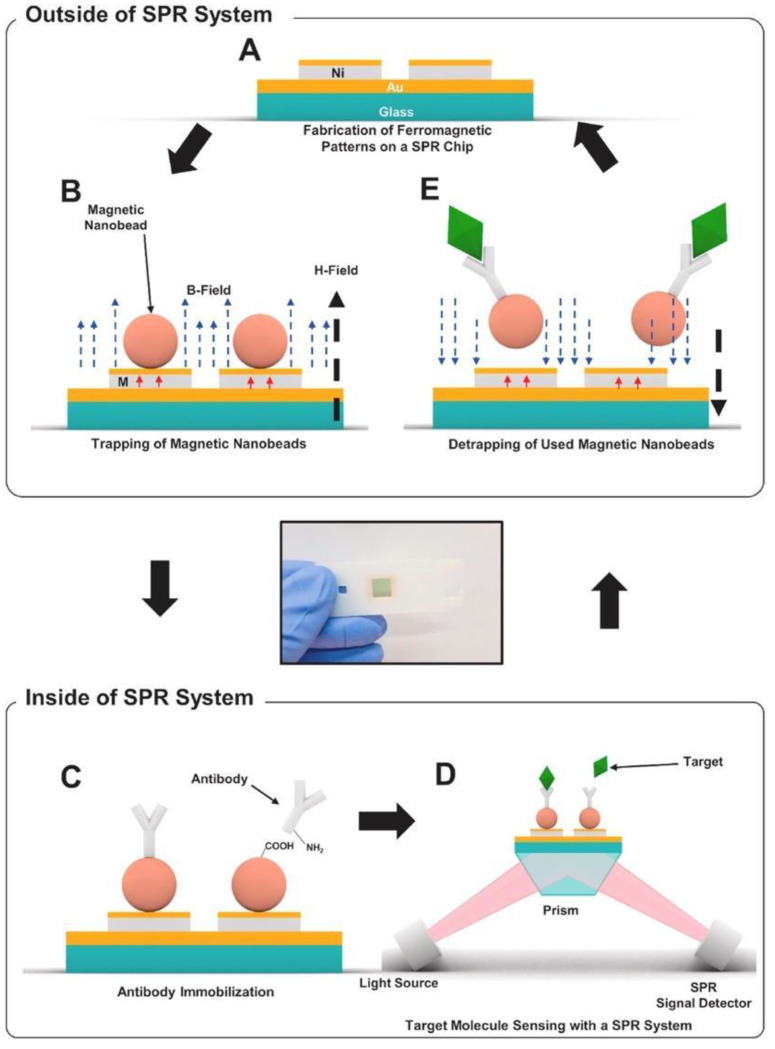
Schematic diagram of reusable SPR biosensor chip: (**A**) SPR chip with ferromagnetic nickel patterns, (**B**) trapping of magnetic particles on the SPR chip through an external magnetic field (H field, 150 mT, black dotted arrow). Magnetization of the ferromagnetic Ni patterns are shown with the red arrows. (**C**) immobilization of antibodies on magnetic particles using EDC-NHS coupling in a conventional SPR system, (**D**) detection process of target molecules, (**E**) removal of magnetic particles with an external magnetic field in the opposite direction to that for trapping. Reproduced with permission from [72]. Copyright © 2020 Elsevier.

As effective and simple methods are needed for virus detection, Yoo et al. designed a reusable magnetic SPR sensor chip for H1N1 influenza virus detection in a conventional SPR sensor. They used ferromagnetic patterns on an SPR sensor chip to prepare a layer of magnetic particles and a solid substrate for SPR sensing [72]. They demonstrated a sensor platform which enables repetitive use of the SPR chip by removing magnetic particles at the end of an experiment using external magnetic fields without the need for antibody-modifying processes. Figure 8 shows the schematic of virus detection using antibodies conjugated to magnetic beads on the substrate. Figure 8A,B depict the ferromagnetic nickel patterns and trapping of magnetic particles on the SPR chip, and Figure 8C schematizes the antibody immobilization on magnetic particles using EDC-NHS coupling. Figure 8D,E show the detection of target molecules and then the removal of the magnetic particles with an external magnetic field, respectively. The magnetic particles on the sensor chip surface are removed using strong external magnetic fields so that the aggregation of magnetic particles on the sensor surface is reduced. The nucleoprotein (NP) of H1N1 influenza virus was applied to the SPR sensor at concentrations between 300 ng/mL and 10 μg/mL. A larger increase in the SPR signal was reported with a higher concentration of the NP solution. This study showed the use of a single reusable SPR chip for the detection of NPs of H1N1 influenza virus for more than seven times without drastic signal degradation. The cost of the SPR sensing was significantly reduced by reusing the SPR chip repeatedly.

The receptor–analyte interaction occurring on the surface of plasmonic biosensors is also monitored using localized surface plasmon resonance (LSPR). Unlike SPR, LSPR is formed by a light wave absorbed within conductive nano-plasmonic materials that are smaller than the wavelength of the incident light [58]. Owing to the enhanced signal amplification achieved by nanomaterials that have specific optical, electrical, and magnetic features, a low limit of detection can be obtained. While the incident light interacts with the metallic nanoparticles (NPs) of the surface, a strong localized EM field generated around these nanostructures enables a strong peak in the course of the absorption spectrum collection at the resonance [73]. The height of the LSPR peak and the corresponding wavelength are affected by not only the sensing medium but also the material type, size, and shape of the plasmonic NPs. The utilization of the nanoparticles for the decoration of the chip surface also provides a large surface area to immobilize a high number of bioreceptor molecules, increasing the sensitivity and specificity of the sensing technique. Several metallic nanostructures, such as nanospheres, nanofibers, nanorods, nanoshells, and nanowires, can be used to fabricate sensing surfaces [74]. The dimensions and the shape of these nanostructures directly affect their plasmonic properties (scattering and absorption ratio, resonance wavelength) [75]. Two main drawbacks of SPR are circumvented with LSPR: first, temperature sensitivity is not an issue for LSPR since the method depends on a simple absorbance measurement; second, less time is required for the whole binding assay due to the faster spread of the analyte to the increased surface area of the NPs compared with a metallic film [76,77]. On the other hand, the response generated by non-specific binding during the analyte incubation and the refractive index variation is the major drawback of LSPR, limiting the applicability and effectiveness of the sensor, especially for the detection from complex samples [78]. Over the last decade, there was a significant increase in the number of nanomaterial-based sensing techniques developed for viral diagnosis.

**Figure 9:**
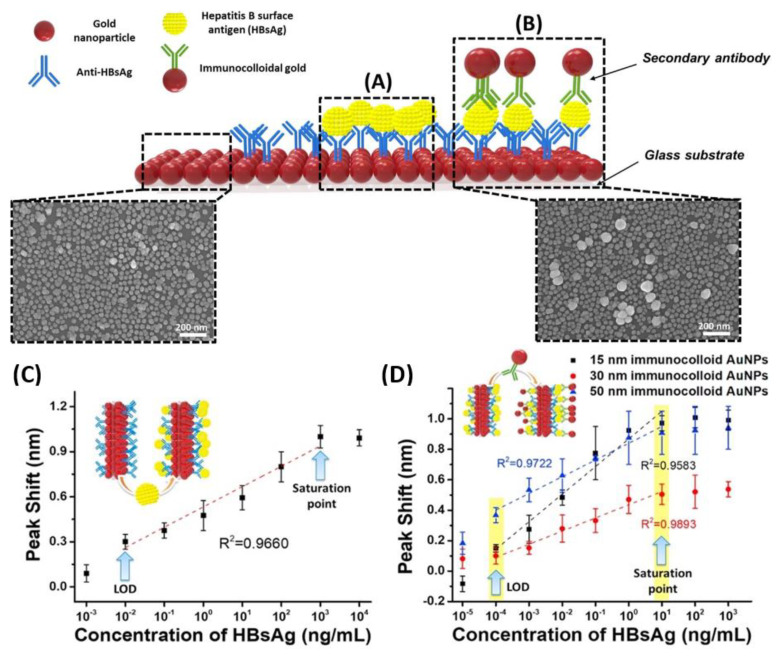
Diagrammatic of LSPR biosensor chip surface. AuNPs are arrayed on the glass substrate. (**A**) LSPR sensing chip surface for single-assay format; (**B**) LSPR chip for modified hetero-assembled AuNP sandwich immunoassay using immune colloidal AuNPs; (**C**) detection of HBsAg using LSPR single-assay format; and (**D**) modified hetero-assembled AuNP sandwich immunoassay format using immunocolloid AuNPs. Reproduced with permission from [79]. Copyright © 2018 Elsevier.

Kim et al. designed a unique structure that used gold nanoparticles (AuNPs) to develop a highly sensitive method for the hepatitis B surface antigen (HBsAg) detection [79]. They designed a single-layered LSPR chip format via antigen–antibody reaction-based detection symmetry using AuNPs. The virus was sandwiched between two different sizes of AuNPs on a glass substrate fabricated with AuNPs. In their study, two AuNPs in close proximity repulse each other in a plasmon resonance state in the presence of the virus, resulting in a stronger peak shift effect than that in the non-sandwich state. A diagrammatic of the LSPR biosensor chip is shown in Figure 9A,B. They showed that implementation-based systems are affected by the particle size in LSPR system. Different concentrations of HBsAg from 1 pg/mL to 1 μg/mL were applied in the single assay LSPR chip format. As shown in Figure 9C, a 10 pg/mL limit of detection value was obtained (Figure 9D). They fabricated a modified detection format to further improve the detection limit by fixing a secondary antibody to the AuNP monolayer, which achieved a 100-times sensitive detection. They showed highly sensitive detection of HBsAg, at 100 fg/mL within 10–15 min, using a novel sandwich immunoassay LSPR chip. Furthermore, the increase in the HBsAg concentration directly caused an increase in the LSPR signals.

**Figure 10:**
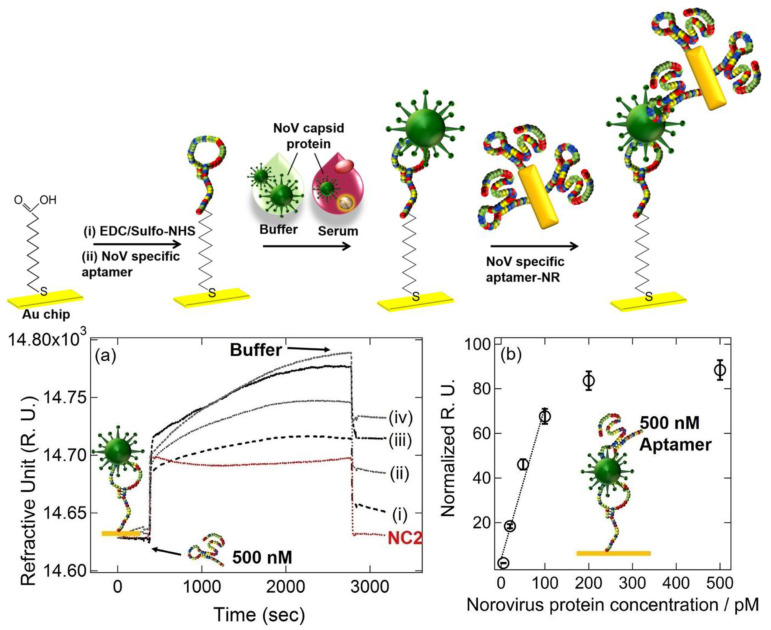
**Top**: The schematic illustration of the aptamer–aptamer sandwich assay for NoV capsid protein detection. **Bottom**: (**a**) Representative SPR sensorgrams for 20–500 pM NoV protein concentrations ((i) 20 pM, (ii) 50 pM, (iii) 100 pM, and (iv) 500 pM) when 500 nM DNA aptamer I was used, (**b**) Normalized RU responses for NoV protein sensing. Reproduced with permission from [80]. Copyright © 2018 Elsevier.

Kim et al. developed a gold-nanorod-enhanced surface sandwich assay for norovirus (NoV) capsid protein detection via a novel pair of aptamers, in conjunction with SPR [80]. They used four different DNA aptamer sequences that were known to be specific for the NoV protein to find the strongest binding constant. The aptamer II sequences were covalently bonded onto a chemically modified thin Au chip surface. For the formation of the surface sandwich complex, the NoV-specific aptamer was attached to the surface of the SPR chip, which was modified via 1-Ethyl-3-(3-dimethylaminopropyl)-carbodiimide hydrochloride and N-(hydroxy-sulfosuccinimide) solution. Then, the NoV capsid protein and gold-nanorod-enhanced aptamer were adsorbed. The authors reported a 50 aM limit of detection value for the NoV capsid protein after flowing different concentrations of NoV protein solutions over the aptamer-modified chip. The NoV capsid protein concentrations were also analyzed in human serum samples. The schematization of the aptamer–aptamer sandwich assay strategy and representative SPR sensorgrams are shown in Figure 10.

### 2.4. Virus Detection with Surface-Enhanced Raman Scattering

Surface-enhanced Raman scattering (SERS)-based sensing platforms rely on the amplification of the Raman response of an analyte molecule absorbed on the nanostructured noble metal substrate. The generation of a new complex between the analyte and metal surface causes modification of the adsorbate polarizability and EM enhancement by improving the re-emitted Raman scattering coming from the analyte and local incident field on the analyte [81,82]. SERS-based sensing gained attention over the last few decades due to its advantages: (i) high sensitivity, (ii) capability for multiplex sensing, (iii) applicability as a POC device, and (iv) laborless sample preparation [83]. Although Raman spectroscopy is a useful tool for analyte determination by providing fingerprints such as a spectrum for complex samples, its inherently weak signals limit its use for diagnosis. However, in SERS technology, the limitations of the weak signal of Raman-active material are overcome by enhancing the EM field by using metallic nanostructures. The development of high-sensitivity SERS sensors with an advanced EM field is carried out by optimizing the design of plasmonic nanostructures [73]. The main advantages of SERS technology are the specific analyte determination even at very low concentrations without sample pre-treatment and its applicability as a POC device [84]. On the other hand, the main challenge that limits the reproducibility of the SERS signal is the signal-reducing degradation in the substrate over time due to the requirement for close contact between the analyte and the amplification surface [85]. SERS-based sensors can be classified as direct and indirect. While the direct method relies on the detection of the spectrum of an analyte, in the indirect technique that is constructed in sandwich format, SERS signals are obtained from the reporter molecule, not the analyte. To differentiate the spectral data of an analyte in the direct technique, the main component and linear discriminant analysis must be performed by comparing the samples of patients and healthy individuals [86]. In the indirect technique, the sensitivity of the method is significantly increased at ultra-low concentrations by using an immunoassay format to detect the analyte. In order to meet the growing need for accurate and rapid virus detection, multiplex immunoassay-based SERS has become prominent due to the limitations of PCR-based techniques that depend only on genetic material for testing [87].

**Figure 11:**
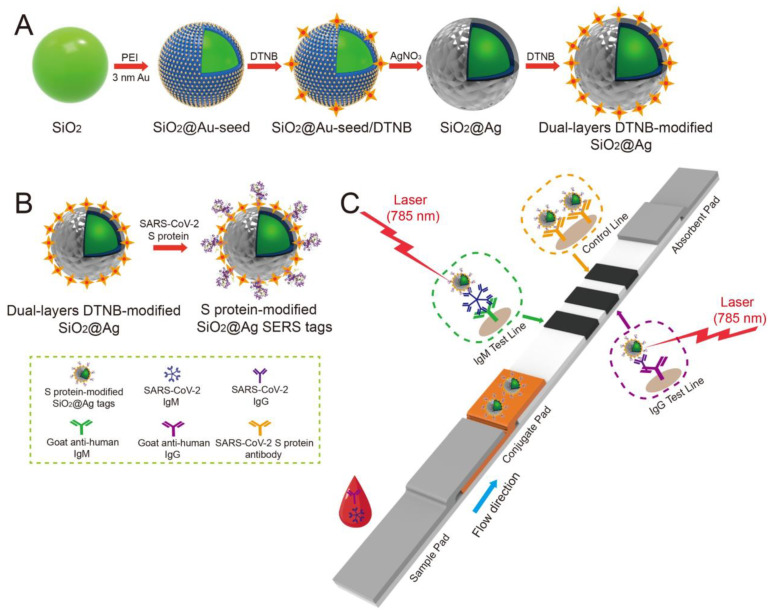
(**A**) Schematic of the dual-layer DTNB-modified SiO_2_–Ag NPs. (**B**) Schematic of the preparation of the SARS-CoV-2 S-protein-modified SiO_2_–Ag SERS probes. (**C**) Simultaneous highly-sensitive anti-SARS-CoV-2 IgM/IgG identification using the SERS-LFIA strip. Reproduced with permission from [88]. Copyright © 2021 Elsevier.

Liu et al. used SERS-based lateral flow immunoassay (LFIA) to detect COVID-19 at the POC [88]. They used Raman molecules to functionalize dual layers of a silver shell on SiO_2_-core NPs as SERS tags. Anti-human IgM and IgG were immobilized onto two test lines of the strip to capture the formed SiO_2_–Ag–spike (S) protein–anti-SARS-CoV-2 IgM/IgG immunocomplexes. The author used a 785 nm excitation wavelength with 10.0 mW laser power. The limit of detection value was 1 pg/mL of the S-protein antibody, which was 800 times lower than that of standard gold-NP-based LFIA for IgM and IgG detection. A schematic of the dual layers of the Raman reporter molecule 5,5′-dithiobis-2-nitrobenzoic acid (DTNB) modified silica–Ag NPs (SiO_2_–Ag) and SERS-LFIA test strip is shown in Figure 11. The outer Ag shell was coated on the SiO_2_ core by the reduction of Ag+ on the Au seeds. As for the optical properties, the change of the UV–vis spectrum of SiO_2_–Ag NPs was reported after Ag shell formation. The absorption peak of SiO_2_–Au seed NPs was centered at 588 nm, while no obvious absorption peak of SiO_2_ NPs and SiO_2_–PEI (polyethyleneimine) NPs was displayed. The transversal and longitudinal plasmon resonances of the SiO_2_–Ag NPs were shown at 371 and 769 nm, respectively, from the Ag shell coating. They revealed that the results showed a high accuracy and specificity for patients with SARS-CoV-2 infection using the designed method.

**Figure 12:**
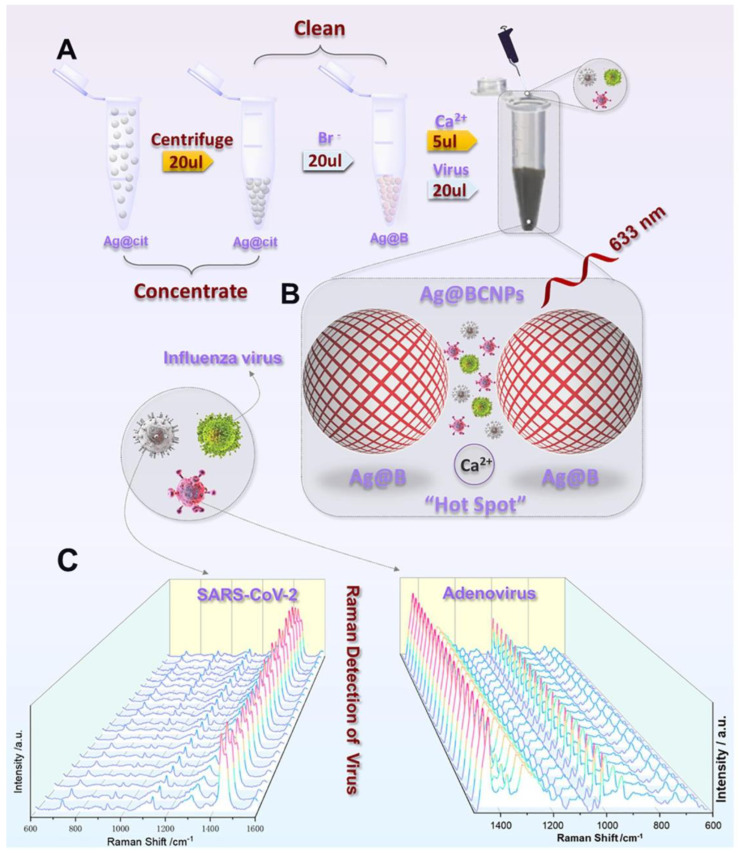
(**A**) Schematic showing the silver-enhanced substrate preparation and virus detection with the SERS technique. (Ag@cit: Silver NPs formed as a result of reduction of citrate; Ag@B: AgNPs with bromide ion; Ag@BCNPs: Ag@B with acetonitrile and calcium ions added). (**B**) Schematic illustration of the interaction between the viruses and the “hot spots” produced by the Ag-enhanced substrate. (**C**) SERS spectra collected from 20 random groups of SARS-CoV-2 (10^4^ PFU/test) and HAdV (10^5^ copies/test) samples using the Ag@BCNP-based method. Reproduced with permission from [89]. Copyright © 2022 Elsevier.

Another new SERS detection system was developed by Zhang et al. They used citrate to reduce AgNPs and added acetonitrile solvent to form a “hot spot” suitable for the capture of viruses. By adding Ca^2+^ and the virus sample together, they detected the human adenovirus and SARS-CoV-2 without the use of a marker [89]. They showed an LOD of 100 PFU/test for these viruses within 1 to 2 min using machine learning techniques. Figure 12 shows the experimental process where AgNPs were modified by bromide ions and acetonitrile, and the generation of hot spots with Ca^2+^ addition.

### 2.5. Optical Resonators for Virus Detection

Optical resonator-based sensor systems have recently attracted significant attention as a powerful tool for detecting a range of biological and chemical analytes with high sensitivity and specificity [90]. These sensors measure the spectral changes in the resonant frequency of an optical cavity when the analyte is introduced into the cavity. The main principle of an optical resonator sensor is based on detecting light-intensity changes induced by changes in the refractive index of the medium surrounding the resonator. The resonator consists of a thin film layer or a ring resonator that supports resonant modes, which are excited by a laser beam. The resonant modes of the resonator are highly sensitive to changes in the refractive index of the surrounding medium. When a target molecule or virus binds to the resonator surface, it causes a change in the refractive index which is detected as a shift in the resonator’s resonant frequency [91].

The basic design of an optical resonator sensor consists of a high-quality factor (Q-factor) resonator, such as a microdisk or microring, and a waveguide coupled to the resonator. The resonator acts as a sensitive transducer capable of detecting changes in the refractive index of the surrounding environment caused by the analyte binding to the resonator’s surface. The change in the resonant frequency is then measured by monitoring the light transmitted through the waveguide. Some of the most common types of optical resonators are Fabry–Perot cavities, whispering gallery mode (WGM) cavities, photonic crystal cavities (PC), and plasmonic resonators [92,93].

For biological particle detection, optical resonators such as microspheres and microtoroids have been used to detect individual virus particles of about 100 nm in a label-free format [94,95]. WGM cavities are highly efficient optical resonators with high Q-factors, allowing for the detection of very small changes in the refractive index of the cavity, and therefore making them useful for various sensing applications. He et al. developed a WGM microresonator using frequency splitting in a microlaser and showed the detection of the influenza A virus on this sensor [96]. Their method relies on measuring the changes in the beat frequency as an ultra-narrow emission line from a WGM microlaser is split into two modes due to nanoparticle binding. Before the nanoparticles arrive, there is a single laser mode with constant laser intensity. The lasing mode splits into two modes when the first nanoparticle binds, generating a beat note with a frequency that is equal to the frequency difference between the two modes. Using this approach, they could detect sizes as small as 15 nm for polystyrene nanoparticles and 10 nm for gold nanoparticles, as well as the influenza A virus. However, this system was tested with purified nanoparticle and virus solutions and has yet to show multiplexed virus detection from complex biological systems.

**Figure 13:**
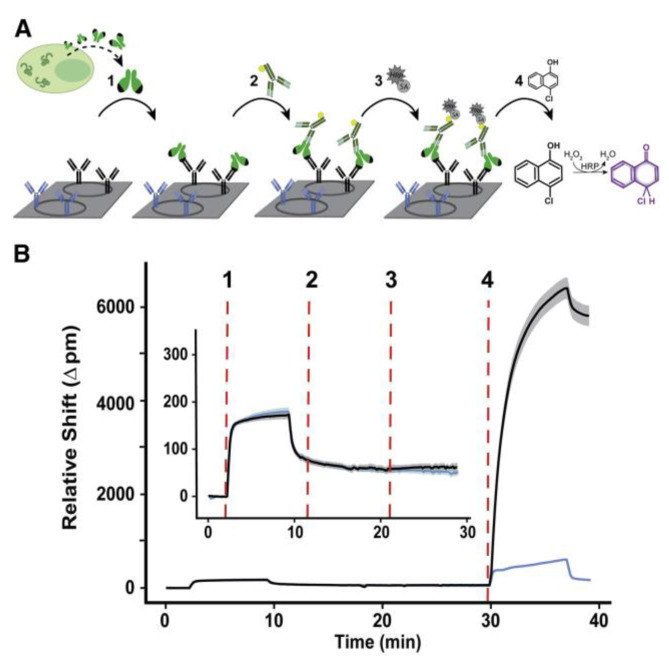
Schematic of microring resonator-based detection of sGP: (**A**) Steps of sGP detection using the microring resonator. (1) Binding of sGP in the serum sample to the specific antibodies immobilized on the microring surface (black). Blue antibodies represent non-specific antibodies as a negative control. (2) Detection of the captured sGP using secondary antibodies, which are biotinylated panfiloviral antibodies. (3) Addition of streptavidin horseradish peroxidase to detect the sandwich complex. (4) Enzymatic reaction. (**B**) Corresponding microring traces for steps 1–4 in (A). The solid line represents the average response of 8–12 replicates, while the surrounding halo corresponds to the spread of individual rings. Inset shows the tracing for steps 1–3. Reproduced with permission from [99]. Copyright © 2022 Elsevier.

As another type of resonator-based sensor, ring resonators have been preferred owing to their unique potential to be coupled in high-throughput arrays efficiently for multiplexed analysis [97,98]. The rapid detection of an Ebola virus (EBOV) biomarker with optical microring resonators was performed by Qavi et al. [99]. Soluble glycoprotein (sGP) is the primary product of the glycoprotein (GP) gene of the EBOV, which is a nonstructural secreted GP that is expressed from the unedited RNA transcript. There are several roles sGP appears to play in EBOV pathogenesis; therefore, it is useful to be utilized as a biomarker in virus detection. In this study, the authors developed a sensor by adapting a silicon photon microring resonator platform to detect EBOV sGP (Figure 13). They used an HRP-dependent sandwich immunoassay to increase the sensitivity and specificity of the assay. The sensitivity is increased due to the precipitate localization on the sensor surface as a result of the enzymatic reaction, providing signal amplification. The sensitivity is enhanced through the use of a secondary antibody since any non-specifically bound molecules on the surface would not be recognized by the secondary antibody. The microring resonator sensor detected sGP in under 40 min with an LOD of 1.00 ng/mL in serum, a much higher analytical sensitivity than the ELISA tests.

**Figure 14:**
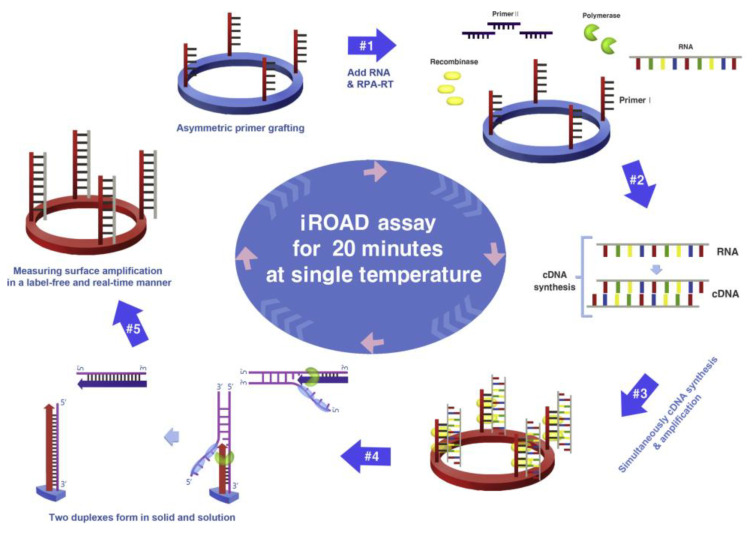
Schematic representation of iROAD assay, which involves an isothermal RNA amplification and label-free detection. (1) iROAD chip preparation by forward primer grafting on the sensor. (2) Addition of the recombinase polymerase amplification (RPA) reagents, reverse primers, and extracted RNA. (3) cDNA synthesis from the RNA template using RT-RPA. (4) Binding of recombinase–primer complexes to cDNA for strand exchange. When the displaced strand makes a D-loop by gp32 (sky blue), primers on the microring surface are extended by polymerase (light green). (5) Formation of two duplexes in solid and solution. The amplification is detected by measuring the wavelength shift on the microring resonator for 20 min. Reproduced with permission from [100] Copyright © 2017 Elsevier.

Koo et al. reported an isothermal, label-free, one-step RNA amplification and detection system, termed iROAD, for the diagnosis of respiratory diseases based on silicon microring resonators [100]. Figure 14 shows the chip fabrication and functionalization for the assay. The iROAD provided an example of a one-step viral RNA amplification/detection assay for rapid analysis (<20 min). They obtained an LOD for the iROAD assay that is ten times lower than that of the real-time reverse transcription-PCR method. The authors tested the iROAD system on 63 human respiratory samples and confirmed its utilization for clinical use as a more robust operation by using an array of microrings for multiplexed detection.

Optical resonators offer several advantages over traditional optical devices, including high sensitivity, selectivity, and miniaturization. Optical resonators can be designed to have a very high Q-factor, which allows for the efficient coupling between the optical signal and the surrounding environment. However, optical resonators also have some limitations, including sensitivity to temperature and fabrication complexity.

### 2.6. Interferometry-Based Sensor Platforms

Viruses are difficult to detect using conventional light microscopy, which mostly relies on measuring the light scattered by the imaged objects. This is due to their small size (typically 20–300 nm in diameter) and low contrast. The light–particle interaction for small-sized particles can be represented by an induced dipole. The strength of an induced dipole is directly proportional to the polarizability of the particle, which can be given as

(1) $$\alpha =4\pi \varepsilon_{0}R^{3}\frac{\varepsilon_{p}-\varepsilon_{m}}{\varepsilon_{p}+2\varepsilon_{m}}$$

where *R* is the radius of the particle and *ε_p_* and *ε_m_* are the permittivity of the particle and medium, respectively. The optical techniques that detect the scattered light intensity generate a signal proportional to |*E_s_*|^2^, which scales with |*α*|^2^; thus, *R^6^*. Therefore, the scattering signal recorded at the detector drops below the shot-noise limit for small particles. On the contrary, interferometric imaging utilizes a strong reference beam (*E_r_*) that interacts with the weak scattered fields (*E_s_*) from the particle and modifies the intensity obtained at the detector as

(2) $$I\propto {\left|E_{r}+E_{s}\right|}^{2}\propto {\left|E_{r}\right|}^{2}+{\left|E_{s}\right|}^{2}+2\left|E_{r}||E_{s}\right|{cos\theta }_{rs}$$

where $\theta_{rs}$ is the phase angle difference between the reference and scattered fields. As the particle size becomes smaller, the scattered field, the second term in Equation (2), becomes very small compared to the other two terms representing the reference field and the interference signal. Once the reference field is subtracted, the signal recorded at the detector is proportional to the multiplication of the reference and scattered fields, and thus proportional to *R*^3^ instead of *R*^6^. As a result, interferometric imaging makes it possible to detect smaller particles and a higher dynamic range of particle sizes. Due to these advantages, interferometry has been utilized for both ensemble-based measurements and single-nanoparticle detection in previous studies [35]. One example of ensemble-based measurement techniques is biolayer interferometry (BLI), a label-free, real-time characterization technique for biomolecular interactions [101]. In this technique, a fiber optic biosensor is used to illuminate the sensor area with white light, and the resulting shift in the wavelength of the reflected light is recorded. Although this technique was shown to perform antibody detection with a similar sensitivity to ELISA, there are some disadvantages associated with it, such as the inability to detect single nanoparticles and signal jumps as the new solutions are introduced to the well [102]. Interferometry-based nanoparticle imaging techniques have also been developed for single-virus detection [103,104]. However, these techniques use cost-inefficient lasers as the light source and can be time-consuming due to their small measurement area.

**Figure 15:**
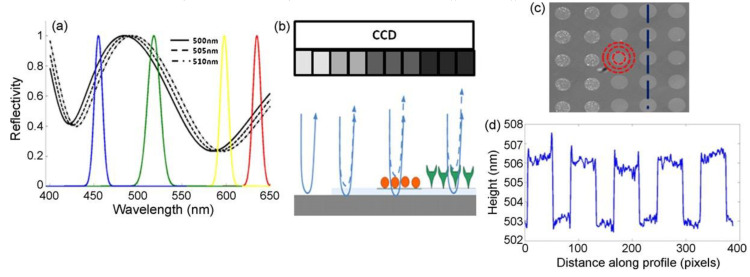
Principle of detection for IRIS: (**a**) shows the shift of the reflectivity curve due to 5 nm step increases in biofilm thickness on the chip surface. The reflectivity curve is sampled by using 4 different-wavelength LEDs shown by the colored Gaussians. (**b**) Schematic of the sensor’s imaging path illustrating biomass accumulation and associated grayscale intensity changes on the CCD camera. (**c**) Example of the sensor’s surface with an array of protein spots. (**d**) Surface height profile along the blue dashed line in (**c**) across spots. Reproduced with permission from [105]. Copyright © 2021 Elsevier.

Interferometric reflectance imaging sensor (IRIS), developed by Ozkumur et al., is a label-free biosensor that can probe bimolecular interactions on a silicon/silicon dioxide (Si/SiO_2_) substrate in a multiplexed microarray format [105,106]. The detection mechanism of IRIS depends on obtaining the interference signature of the reflected light from the Si/SiO_2_ substrate and measuring the optical thickness of the top layer (Figure 15). The substrate is a silicon chip with a thermally grown oxide layer that is spotted with capture probes that are specific for the target molecule. The binding of target molecules in the solution to the surface causes a height increase in the transparent oxide film, which in turn changes the optical path length difference (OPD). The intensity of the reflected light at a given wavelength is determined by the OPD between the top of the biomass layer and the Si/SiO_2_ interface. The thicker the biomass layer becomes, the higher the OPD becomes, and this increase in the OPD leads to a shift in the spectral reflectivity curve (Figure 15a) and a change in the reflected light intensity at a specific wavelength. In the IRIS optical setup, four LEDs with different wavelengths (455, 518, 598, and 635 nm) illuminate the substrate sequentially and the intensity of the reflected light is recorded with a CCD to generate an intensity image of the chip surface. Then, each pixel in this intensity image is fitted to the reflection function from which the thickness of the transparent film (oxide layer plus any biomass layer) is obtained. The calculated thickness difference for a given spot before and after analyte incubation is converted to biomass density using simple conversion factors, and an average mass density is calculated from replicate spots [107].

**Figure 16:**
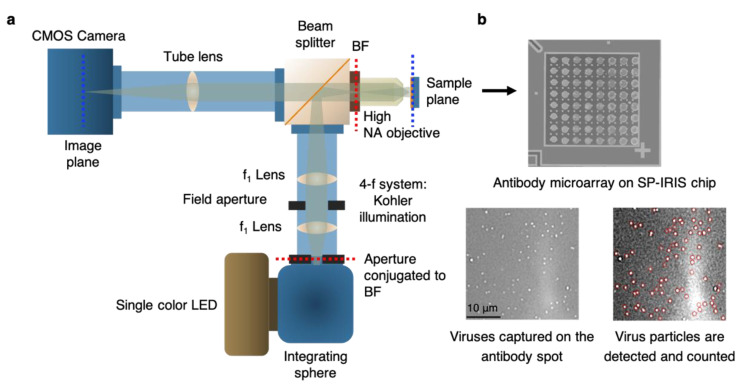
Optical setup of SP-IRIS and its application to virus detection. (**a**) Illustration of optical setup of SP-IRIS platform (BF: back focal plane). (**b**) Top part is an example of SP-IRIS microarray chip with antibody spots. The image was taken with the low-magnification modality of the IRIS system. Each antibody spot is about 150 μm in diameter. Bottom images are zoomed-in antibody spots from an SP-IRIS image after incubation with the specific virus sample. Captured virus particles appear as white dots, and they are detected (red circles) and counted using custom software. Reproduced with permission from [115]. Copyright © 2023 Spring Nature.

IRIS is shown to be a versatile platform for monitoring protein–protein [108], [109], DNA–DNA [110], and DNA–protein interactions [111], with applications in cytokine detection, the identification of single nucleotide polymorphisms (SNPs), the study of DNA-binding proteins such as transcription factors, and antibody affinity measurements. IRIS was also used to detect intact vesicular stomatitis virus (VSV) particles with a detection limit close to 10^5^ PFU/mL as well as internal viral proteins, such as nucleocapsid and matrix proteins, by lysing the viruses with detergent [112]. The IRIS platform was later modified to generate a digital detection modality to allow for the visualization and counting of single nanoparticles. This platform, referred to as single-particle IRIS (SP-IRIS), is composed of a single-wavelength LED (525 nm) for illumination of the substrate, a high-numerical-aperture (NA = 0.8) objective to obtain a high-spatial-resolution image, and a CCD camera [113]. The thickness of the oxide layer was adjusted to optimize the interference of the particle scattered field with the reference field. The schematic of the optical setup for SP-IRIS is shown in Figure 16a.

For detecting biological nanoparticles such as viruses, an array of high-affinity capture probes is generated on the surface that can selectively bind to the target virus. Capture probe immobilization is achieved using a 3D copolymer coating that provides NHS groups for the covalent attachment of amine-containing biomolecules [114]. When virus particles bind to the capture antibodies, scattered light from the particles interferes with the reference field reflecting from the Si/SiO_2_ interface, which enhances the signal detected on the CCD camera. The particles captured on the chip surface are bright dots in the recorded image (Figure 16b). SP-IRIS takes images of the spots in a microarray. Then, these spot images are analyzed using custom software that finds particle-associated intensity peaks that correlate with a Gaussian profile. A forward model is applied to associate the background normalized intensities (contrast) of the particles with the particle size [113]. Therefore, the diffraction-limited dots with an expected size range are selected for a given spot using a Gaussian filter (red circles in Figure 16b) and counted to obtain the number of virus particles bound to the spot. The pre-incubation particle count is subtracted from the post-incubation particle count, and the net virus count is divided by the spot area to obtain the bound virus density (number of particles per mm^2^). The single-particle detection ability of SP-IRIS offers a significant advantage over ensemble-based methods, such as BLI, where many binding events need to occur to record a signal above the background noise. The high sensitivity achieved by single-virus counting, when combined with the on-chip multiplexing ability, renders SP-IRIS an attractive platform for viral diagnostics applications.

### 2.7. Virus Diagnostics Applications of SP-IRIS

SP-IRIS can count and size each nanoparticle bound to capture probes on the sensor surface over a large sensor area, orders of magnitude larger than other virus-imaging techniques such as electron microscopy. It allows for a large range of nanoparticle detection, including natural nanoparticles (e.g., viruses) and synthetic nanoparticles (e.g., gold nanospheres, gold nanorods) in a highly-multiplexed microarray format. So far, SP-IRIS has been shown to detect many different biological targets, such as viruses [116], allergen-specific antibodies [117], extracellular vesicles [118], bacteria [119], and microRNA [120]. When the target is a nanoparticle itself, such as viruses, the detection can be performed directly without using any secondary labels. If the biomolecule being searched for is below the size limit of SP-IRIS (~30 nm), the target binding can be monitored using specific detection probes attached to nanoparticle barcodes such as gold nanoparticles. Since this review’s main topic is solid-phase optical virus detection techniques, we will focus on the SP-IRIS studies demonstrating this application.

**Figure 17:**
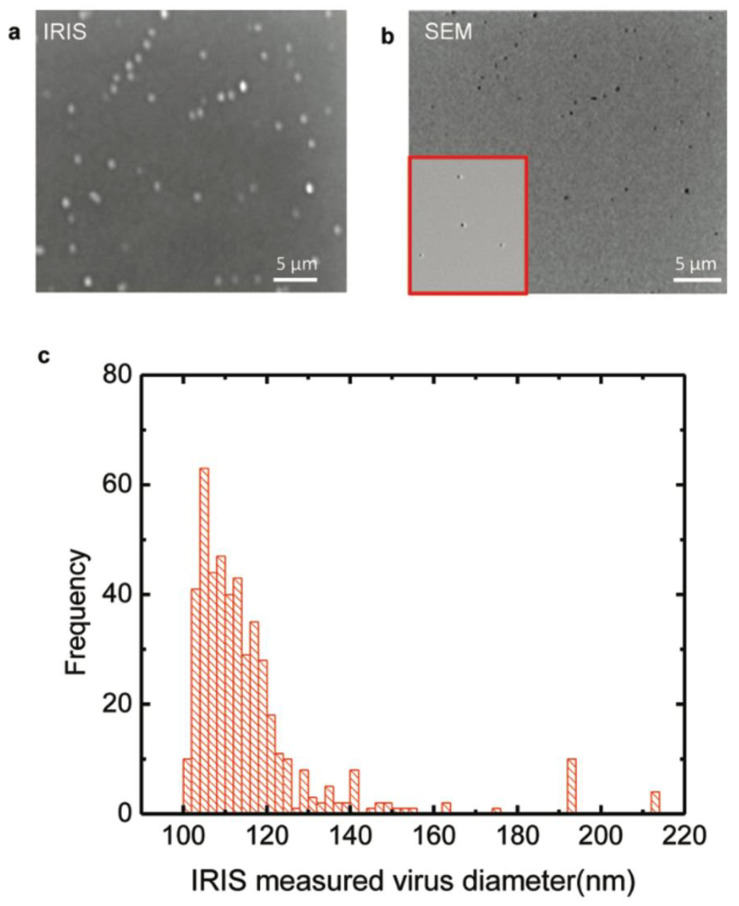
H1N1 virus detection and sizing: (**a**) SP-IRIS image of immobilized virus on the surface with the same field of view as the SEM image; (**b**) SEM image of immobilized virus on the surface; (**c**) measured size distribution of immobilized virus using SP-IRIS. The mean size of the H1N1 was measured to be 116 ± 17 nm. Reproduced with permission from [113]. Copyright © 2010 American Chemical Society.

The study by Daaboul et al. was the first report to show SP-IRIS as a virus-detection platform by demonstrating the detection and sizing of individual H1N1 viruses [113]. In this work, H1N1 viruses were immobilized on the sensor surface and imaged using both SP-IRIS and scanning electron microscopy (SEM) for the exact same field of view. Their results showed a one-to-one correspondence between SP-IRIS and SEM images, confirming that the particles observed in the SP-IRIS system are virus particles, proving the sensor’s ability to detect individual viruses (Figure 17). In the same work, using the forward model mentioned before, they measured the mean size of the H1N1 particles as 116 nm with a size distribution of 17 nm, which is in good agreement with the reported H1N1 virus size in the literature.

**Figure 18:**
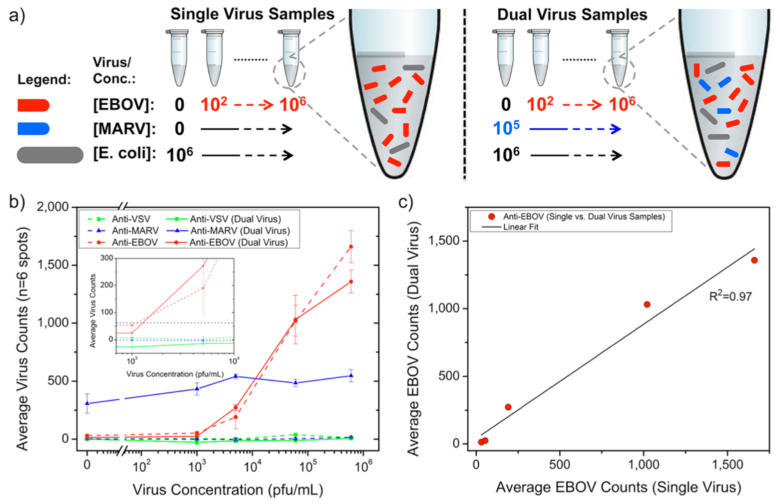
Duplexed detection of Ebola- and Marburg-pseudotyped VSVs in serum and bacteria-containing samples. (**a**) Schematic of virus sample preparation. Both single- and dual-virus samples were prepared in serum containing 10^6^ CFU/mL *E. coli* K12. (**b**) Anti-EBOV antibody spots showed an exponential response as the rVSV-EBOV concentration increased. Single and dual virus samples generated nearly identical virus counts on anti-EBOV spots for the same rVSV-EBOV titers, whereas the addition of Marburg pseudotype at a constant concentration gave a steady signal on anti-MARV antibody spots for all rVSV-EBOV concentrations. The inset displays an expanded view of virus concentrations around 10^3^ PFU/mL; the dashed line represents the detection threshold calculated from the mean plus three standard deviations for the anti-EBOV spots incubated in the FBS alone. For (**b**), the lines connecting the data points are given only to guide the reader’s eye between single- and dual-virus sample responses. (**c**) The response observed between the anti-EBOV spots in single- and dual-virus samples was similar, as shown by the linear regression fit to a scatter plot of single-virus sample type against the dual sample. Reproduced with permission from [116]. Copyright © 2014 American Chemical Society.

Following the first virus-detection demonstration with immobilized H1N1 virus, SP-IRIS was shown to perform sensitive and multiplexed detection of whole viruses from serum and blood samples [116]. For this work, Daaboul et al. used genetically engineered vesicular stomatitis virus (VSV) pseudotypes that express surface glycoproteins of Ebola and Marburg viruses (rVSV-EBOV and rVSV-MARV, respectively) (Figure 18). They first arrayed EBOV- and MARV-specific antibodies on SP-IRIS chips and incubated the chips with either increasing concentrations of rVSV-EBOV only or the same increasing rVSV-EBOV concentrations in the presence of a constant concentration of rVSV-MARV. The virus solutions were prepared in serum containing 10^6^ CFU/mL *E. coli* K12 to mimic a complex solution environment. Their results showed the specific detection of rVSV-EBOV with increasing virus particles on anti-EBOV spots, whereas anti-MARV spots had a constant signal in dual-virus samples. The limit of detection (LOD) reported for rVSV-EBOV detection from serum and blood was 5 × 10^3^ PFU/mL. A similar level of sensitivity was also reached for rVSV-MARV detection in the same study. This work demonstrated that SP-IRIS has great potential to be used as a viral diagnostic technique with its ability to directly detect the target viruses from complex samples without labeling and complicated sample preparation.

**Figure 19:**
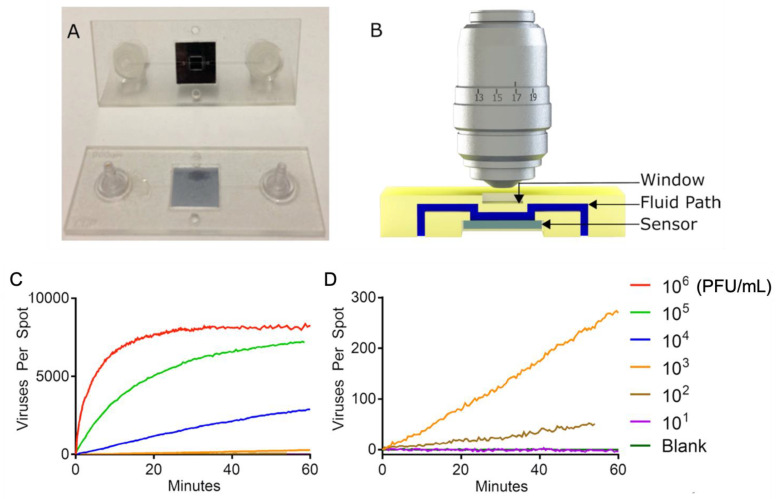
(**A**) Picture of the first-generation polymeric cartridge used for real-time virus imaging. (**B**) Cross-section model that demonstrates the fluidic path (in blue), the sensor (in gray), and the window (in yellow). Image is not to scale. (**C**) Accumulation of viruses being imaged on the sensor for a serial dilution ranging from 1 × 10^6^ PFU/mL down to a blank sample. The high-concentration samples show a very rapid accumulation of viruses followed by the saturation of the sensor. (**D**) Lower concentrations expanded, showing a linear accumulation of viruses and a limit of detection of 100 PFU/mL in less than 60 min. Reproduced with permission from [121]. Copyright © 2016 American Chemical Society.

SP-IRIS was further advanced to implement the ability to visualize virus particles in a liquid environment, rendering the system a real-time imaging platform and eliminating the washing and drying steps (Figure 19). To increase the contrast of the virus particles in the liquid, some changes to the optical setup were made, such as the use of a 40×, 0.9 NA objective and adjustment of the oxide thickness of the sensor chip. In this setup, the SP-IRIS chip is mounted in a disposable active microfluidic cartridge via a pressure-sensitive adhesive, and the cartridge is fixed on the SP-IRIS stage. Scherr et al. reported a 50-fold increase in sensitivity compared to in-air measurements, leading to an LOD of 100 PFU/mL for detecting rVSV-EBOV from serum samples [121].

To demonstrate the applicability of SP-IRIS to POC diagnostics as a rapid detection method, a disposable passive microfluidic cartridge was designed with a multilayer polymer laminate structure and an integrated absorbent paper to establish capillary flow of the sample in the cartridge. This passive-flow integrated SP-IRIS achieved a better sensitivity than ELISA and a commercial rapid antigen test by detecting 10^4^ PFU/mL rVSV-EBOV in less than 20 min [122,123]. A different study by Daaboul et al. demonstrated the usability of SP-IRIS for the detection and characterization of a variety of virus sizes ranging from 40 nm for the Zika virus to 360 nm for the Vaccinia virus and filamentous virus particles such as the Ebola virus [124]. Recently, Yurdakul et al. showed a different modality of SP-IRIS, referred to as single-particle interferometric microscopy, for obtaining shape and size information that will enable in-depth morphological studies of viruses [125]. Collectively, these studies demonstrated the potential of SP-IRIS as a sensitive, fast, and multiplexed virus-detection platform in a label-free and sample-to-answer format.

**Figure 20:**
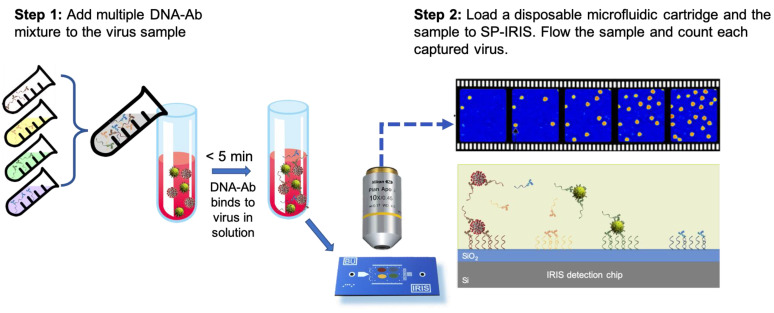
Schematic demonstration of the DNA–Ab conjugate-directed capture of the viruses on SP-IRIS. (**Step 1**) involves mixing different DNA-conjugated antibodies, each targeting a different virus, and adding this mixture to the sample potentially containing a virus. After about a 5 min incubation, in (**Step 2**), the sample is flowed over an SP-IRIS chip that is assembled into a disposable microfluidic cartridge. Virus–DNA–Ab complexes are captured on the chip surface through DNA–DNA hybridization. Free DNA–Ab conjugates bound to the DNA spots are invisible in camera images. Only captured viruses appear as bright dots.

Besides the microfluidics integration and improvements in the optical setup of SP-IRIS, sensor-chip surface chemistry was also studied in an effort to increase the sensitivity of detection. By using a technique called DNA-directed antibody immobilization (DDI), Seymour et al. showed that capture antibodies can be elevated over the surface (~14 nm) through the use of DNA linkers attached to the antibodies [126]. This new surface-preparation technique provided a 16-fold increase in sensitivity for rVSV-EBOV detection for a 15 min incubation period. This improvement is most likely due to the increased accessibility of the antibodies for virus binding and increased functionality due to fewer surface attachment points in the antibody structure. This work was recently extended to a novel approach of mixing DNA–antibody conjugates and the virus sample in the solution phase before incubating the chip (Figure 20). This homogeneous method achieved a slightly better sensitivity than conventional DDI while decreasing the assay time [123]. Other advantages of this approach include a configurable sensor surface, reduced amount of antibody needed for the assay, and long shelf-life of the dried DNA–Ab conjugates.

Table 1 presents some examples of viral diagnostics platforms operating with the optical mechanisms reviewed in this work and compares their performances in terms of linear range, LOD, and assay time. With the recent advances in camera and detector development and with the use of smartphones, fluorescence- and colorimetry-based optical bioassays have become a common choice in POC technologies since they provide sufficient sensitivity in virus detection. They provide the users with the advantages an ELISA assay offers, such as a low limit of detection, quantitative measurement, and applicability with various samples. However, some may require a tedious process for sample preparation, and the assay conditions, such as the temperature and pH of the sample, may affect the results. In addition, the labeling process is time-consuming for both techniques and may require complex steps in some applications.

SPR sensors are commonly reported to have a very high sensitivity and provide simple and real-time detection; however, the bulk effect and low selectivity are the main disadvantages of SPR systems. The main advantages of LSPR systems are ease of operation, fast detection, and insensitivity to the temperature changes, enabling their use in many areas. However, LSPR-based platforms cannot distinguish between different binding events, making them less useful for multiplexed analysis.

The SERS system has gained attention due to its sensitivity, capability for multiplex sensing, and specific analyte determination ability, even at very low concentrations. On the other hand, signal-reducing degradation in the substrate over time due to the requirement for close contact between the analyte and the amplification surface is the main challenge limiting the reproducibility of the SERS signal.

SP-IRIS offers significant advantages compared to the other optical biosensing platforms mentioned in this review. First, SP-IRIS has a comparable sensitivity to SPR, the most commonly used label-free biosensor, while having a higher multiplexing capability, substantially less-expensive substrates, and a shorter analysis time [109]. Moreover, the detection principle of SP-IRIS is immune to the bulk effect, a major problem of SPR-based systems caused by the changes in the refractive index of the solution. SP-IRIS overcomes any background-related effect by imaging only the nanoparticles bound to the surface. Moreover, unlike optical-resonator-based sensors, the SP-IRIS signal is not affected by environmental factors such as temperature changes or the binding position of the particles on the sensor. Thus, SP-IRIS combines a robust and reliable signal-transduction mechanism with high-sensitivity, high-throughput detection in a cost-effective and easy-to-use platform. The challenges related to the surface probe immobilization and the sensor chip shelf-life were overcome by implementing configurable DNA chips, and the assay procedure was greatly simplified by microfluidic integration. These features make SP-IRIS an ideal candidate for rapid and reliable virus diagnostics, especially for POC applications.

## 3. Conclusions

In this review, we discussed several surface-based optical biosensing techniques that are used to detect and characterize viruses. Recent outbreaks revealed the importance of developing sensitive, reliable, rapid, and affordable viral diagnostic platforms. Multiplexing ability is also considered critical, especially in cases where diseases with similar physical symptoms occur at the same time, such as COVID-19 and seasonal flu. In addition, the ideal virus-detection platform should operate with minimum sample preparation in a preferably enclosed environment.

The biggest advantage of SP-IRIS compared to other optical techniques lies in its ability to detect single viruses in a robust and cost-efficient platform. Moreover, recent advancements in SP-IRIS enabled it to work with integrated microfluidic cartridges, eliminating the chip-handling steps. SP-IRIS can also perform sensitive and specific detection from complex media such as blood and serum, decreasing the assay time and complexity. In addition, lately, data processing for SP-IRIS assays has been improved, allowing it to be performed in real time. Therefore, the assay results are available at the end of the sample incubation period (<20 min). All these developments have increased SP-IRIS’s potential to be a virus diagnostic platform that is attractive for both in-clinic and field use.

## Figures and Tables

**Table 1:** Examples of solid-phase optical biosensors using different detection mechanisms, their applications, and assay-performance characteristics.

Platform	Optical Sensing Technique	Application	Linear Range	LOD	Time	References
Porous Au@Pt nanoparticle for metallic nanozyme-catalysis	Colorimetric	S1 protein of SARS-CoV-2	10–100 ng/mL	11 ng/mL	NR	[127]
SiO_2_@Au@QD nanobeads (NBs) labels in lateral flow immunoassay (LFIA)	Colorimetric–Fluorescent dual-mode	IgM and IgG in human serum	10^1^×–10^6^× dilution of serum specimens	1:10^6^ dilution of serum specimens	15 min	[128]
SiO_2_@Au/QD in LFIA	Colorimetric–Fluorescent dual-functional	S1 protein of SARS-CoV-2 and real virus	0.05–1000 ng/mL protein	Colorimetric: 1 ng/mL protein, 7.06 × 10^5^ copies/mL virions, Fluorescence: 33 pg/mL protein, 1.02 × 10^4^ copies/mL virions	30 min	[129]
Wearable microfluidic device with recombinase polymerase amplification (RPA)	Fluorescence	HIV-1 DNA	10^2^–10^5^ copies/mL	100 copies/mL	24 min	[130]
Hydrogel aptasensor embedded with QD fluorescent reporters	Fluorescence	Avian influenza virus (AIV) H5N1	0.4–32 HAU	0.4 HAU	30 min	[131]
Intensity-modulated SPR	SPR	Avian influenza A H7N9 virus	NR	144 copies/mL	NR	[71]
Magnetic particles based SPR	SPR	H1N1 influenza virus	300 ng/mL–10 μg/mL	NR	350 s	[72]
Gold nanoparticles based LSPR	LSPR	Hepatitis B surface antigen	1 pg/mL to 1 μg/mL	10 pg/mL	10–15 min	[79]
Self-assembled plasmonic nanoprobe POC device	LSPR	SARS-CoV-2	1–1 × 10^4^ CFU/mL	1.4 × 10^1^ PFU/mL	10 min	[132]
Gold nanorod-enhanced surface sandwich assay	SPR	Norovirus	20–500 pM	50 aM	NR	[80]
SiO_2_–Ag nanocomposite	SERS-based LFIA	COVID-19 virus	10–0.001 ng/mL	1 pg/mL	NR	[88]
Ag NPs	SERS	SARS-CoV-2	NR	100 PFU/test	1–2 min	[89]
MBSIs@Ag-SERS	SERS	H5N1 influenza virus	5.0 × 10^6^–5.0 × 10^−7^ TCID_50_/mL	5.0 × 10^−6^ TCID_50_/mL	NR	[133]
Opto-fluidic ring-resonator-based sandwich assay	WGM microresonator	M13 filamentous bacteriophage	2.3 × 10^3^–2.3 × 10^10^ PFU/mL	2.3 × 10^3^ PFU/mL	NR	[134]
One-step viral RNA amplification and detection—iROAD	Silicon microring resonator	Influenza A/B, human coronavirus (HCoV)-OC43/229E, or respiratory syncytial virus (RSV)-A/B	2.5 × 10^1^–2.5 × 10^5^ copies/reaction	25 copies/reaction	<20 min	[100]
SP-IRIS	Interferometric imaging	Whole-virus detection (VSV-based model viruses)	10^2^–10^6^ PFU/mL	10^2^ PFU/mL	<20 min	[121,123]
Biolayer Interferometry (BLI)-based antibody detection	Interferometric	Norovirus antibodies	10^2^–10^3^ fold dilution of serum samples	1:10^4^ dilution of serum	10–20 min	[135]
Young Interferometer Sensor	Interferometric	Herpes simplex virus type 1 (HSV-1)	8.5 × 10^2^ to 8.5 × 10^6^ particles/mL	850 particles/mL	1 h	[136]

## Data Availability

Not applicable.
